# Significance and Diagnostic Accuracy of Early S100B Serum Concentration after Aneurysmal Subarachnoid Hemorrhage

**DOI:** 10.3390/jcm9061746

**Published:** 2020-06-05

**Authors:** Baptiste Balança, Thomas Ritzenthaler, Florent Gobert, Caroline Richet, Carole Bodonian, Romain Carrillon, Anne Terrier, Laurent Desmurs, Armand Perret-Liaudet, Frédéric Dailler

**Affiliations:** 1 Hospices Civils de Lyon, Hôpital Pierre Wertheimer, Department of Neurological Anesthesiology and Intensive Care Medicine, 69500 Lyon, France; thomas.ritzenthaler@chu-lyon.fr (T.R.); Florent.GOBERT01@chu-lyon.fr (F.G.); carole.bodonian@chu-lyon.fr (C.B.); romain.carrillon@chu-lyon.fr (R.C.); anne.terrier@chu-lyon.fr (A.T.); frederic.dailler@chu-lyon.fr (F.D.); 2 Inserm U1028, CNRS UMR 5292, Lyon Neuroscience Research Centre, Team TIGER, 69500 Lyon, France; 3 Université Claude Bernard Lyon 1, Centre Lyonnais d’Enseignement par la Simulation en Santé, SAMSEI, 69008 Lyon, France; 4 Hospices Civils de Lyon, Hôpital Pierre Wertheimer, Clinical Chemistry and Molecular Biology Laboratory, 69500 Lyon, France; caroline.richet@chu-lyon.fr (C.R.); laurent.desmurs@chu-lyon.fr (L.D.); armand.perret-liaudet@chu-lyon.fr (A.P.-L.); 5 Inserm U1028, CNRS UMR 5292, Lyon Neuroscience Research Centre, Team BIORAN, 69500 Lyon, France

**Keywords:** S100B protein, subarachnoid hemorrhage, early brain injury, patient outcome assessment

## Abstract

Background: Early brain injuries (EBI) are one of the most important causes of morbidity and mortality after subarachnoid hemorrhage. At admission, a third of patients are unconscious (spontaneously or sedated) and EBI consequences are not evaluable. To date, it is unclear who will still be comatose (with severe EBI) and who will recover (with less severe EBI) once the aneurysm is treated and sedation withdrawn. The objective of the present study was to determine the diagnostic accuracy of S100B levels at hospital admission to identify patients with severe neurological consequences of EBI. Methods: Patients were consecutively included in this prospective blinded observational study. A motor component of the Glasgow coma score under 6 on day 3 was used to define patients with severe neurological consequences of EBI. Results: A total of 81 patients were included: 25 patients were unconscious at admission, 68 were treated by coiling. On day 3, 12 patients had severe consequences of EBI. A maximal S100B value between admission and day 1 had an area under the receiver operating characteristic curve (AUC) of 86.7% to predict severe EBI consequences. In patients with impaired consciousness at admission, the AUC was 88.2%. Conclusion: Early S100B seems to have a good diagnostic value to predict severe EBI. Before claiming the usefulness of S100B as a surrogate marker of EBI severity to start earlier multimodal monitoring, these results must be confirmed in an independent validation cohort.

## 1. Introduction

The 28-day mortality associated with subarachnoid hemorrhage (SAH) is reported to range from 26% to 40% in Europe [1] and half of those who survive sustain irreversible brain damage [2]. Bleeding consequences, such as cerebral edema, intracerebral hemorrhage, mechanical stress, or raised intracranial pressure, as well as re-bleeding or aneurysm treatment complications, are important determinants of poor outcome [3,4,5,6,7]. These first brain insults have been grouped together and termed early brain injuries (EBI). Physicians usually rate EBI at two critical moments: at the first physician contact using the World Federation of Neurosurgical Surgeons (WFNS) grading scale, which is a good predictor of clinical outcome [8]; and 3 days later, once the aneurysm is secured, potential hydrocephalus is treated, anesthesia is withdrawn, and before the period during which delayed cerebral ischemia (DCI) can occur, as the latter impacts prognosis [9,10]. At day 3, patients without any loss of consciousness or neurological deficit, supposed to have no or mild EBI, can be differentiated from unconscious patients suffering severe EBI [9,10]. Since severe EBI increases the risk of DCI, it is important to identify early on those who require multimodal monitoring to detect delayed injury [11,12,13]. Yet, the consequences of EBI may not be assessable by clinical examination, as many patients require sedation before or at admission to the intensive care unit (ICU; e.g., due to respiratory failure, early seizure or loss of consciousness).

Several early markers have been used to rate EBI after SAH. For instance, the presence of a cerebral edema on computed tomography (CT) images, graded using the subarachnoid hemorrhage early brain edema score (SEBES), or the extent of fluid attenuation inversion recovery (FLAIR) hypersignals on magnetic resonance imaging, are predictors of long-term disability and DCI occurrence [6,7,14]. Systemic biomarkers have also been evaluated to rate the burden of brain injury, such as the neuron specific enolase (NSE) following cardiac arrest [15,16]. However, in hemorrhagic stroke, astrocytic markers are better biomarkers of the primary hemorrhagic lesion [17,18,19]. The most studied is S100B, a calcium-binding protein expressed predominantly in astroglial cells. A high serum S100B level during the first days after SAH is also predicative of a poor long-term outcome, and is increased by cerebral infarction [18,20,21]. However, the long-term outcome is the result of both early and delayed injury, and it is unclear whether S100B can be used to rate EBI severity. In the absence of an early biomarker to identify patients who will remain unconscious (i.e., with severe EBI) 3 days after the bleeding, we hypothesized that early S100B serum concentration reflects the extent of EBI caused by the primary hemorrhage.

The primary aim of this study was therefore to evaluate the diagnostic accuracy of S100B to predict the severe consequences of the primary hemorrhage. We chose a pragmatic clinical approach and defined nonconscious patients at day 3 as having severe consequences of EBI. We also explored early S100B diagnostic accuracy to predict the outcome at ICU discharge.

## 2. Experimental Section

This prospective blinded single-center observational study was conducted in the neurological department of anesthesiology and intensive care medicine of the Pierre Wertheimer Hospital (Hospices Civils de Lyon, Lyon, France). S100B measurements were not available to the clinical staff and therefore did not influence therapeutic choice or any outcome assessment. Written and oral information was given to the patients or their relatives. This study is part of the single-center Prospective, Observational Registry of Patient with Subarachnoid Hemorrhage in Neurocritical Care Unit (ProReSHA) which was approved by our local ethics committee (Comité de Protection des Personnes Lyon Sud Est II, n°IRB: 00009118), and registered on clinicaltrials.org (NCT02890004).

### 2.1. Participants

The ProReSHA started in September 2016. Since S100B measurements were available in our hospital on 28 November 2016, the patients of the study reported herein were consecutively included from this date to 23 November 2017. Inclusion criteria were admission for a recent clinical history of SAH with evidence of bleeding on CT and age ≥ 18 years. Exclusion criteria were traumatic SAH, the absence of aneurysm on first angiography, a delay > 36 h between the ictus and ICU admission, and ongoing sedation at day 3.

### 2.2. Outcome

The definition of EBI can vary depending on the method of evaluation (i.e., clinical evaluation, brain imaging, invasive neuro-monitoring, neuroinflammation, electrophysiology) [10]. We chose a pragmatic approach usable at the bedside and tried to differentiate patients without or with mild EBI (e.g., without loss of consciousness) from those with moderate EBI (i.e., with an initial loss of consciousness that will rapidly recover) and those with high EBI who will remain unconscious for several days. Although the WFNS score is used to assess patient severity at first physician contact, it was not designed to monitor clinical changes during the hospital stay. Instead, the Glasgow coma scale (GCS) is used to monitor the neurological state and look for a new neurological deficit after SAH [22]. Therefore, its motor component (M-GCS) on day 3 was chosen as the reference for early outcome, as M-GCS is both widely used and reproducible at the bedside and because, at day 3, patients are expected to have received aneurysm treatment, with or without hydrocephalus treatment, and anesthesia should have been withdrawn. A score < 6 on the M-GCS indicates an unconscious patient who does not respond to an oral command. Based on the M-GCS at ICU admission and day 3, we defined three groups: the EBI-mild group for whom the M-GCS was 6 at arrival and day 3, the EBI-moderate group for patients with an M-GCS < 6 at arrival and 6 on day 3, and the EBI-severe group presenting an M-GCS score < 6 at day 3 regardless of the M-GCS on arrival.

### 2.3. Clinical Evaluation

The physicians and nurses in charge of patients and the clinical data assessors were blinded to the S100B levels. SAH severity was evaluated using the WFNS at first contact with a physician (emergency room or out of hospital) and the GCS at admission to the ICU [8]. The Fisher grade [23,24], the SEBES [14], and the Hijdra score [25] were also collected from the first CT. On day 3, the best motor, eye, and verbal GCS sub-scores were collected from the daily evaluation. Other clinical and laboratory parameters, as well as medications, were available hourly from the IntelliSpace Critical Care and Anesthesia software (Philips Informatique Médicale, Suresnes, France), as well as the presence of sedation and hemodynamic or ventilation support. The assessor of the modified Rankin Scale (mRS) at ICU discharge was also blinded to the S100B levels.

### 2.4. Clinical Management

Patients were managed per international SAH treatment guidelines [2]. Briefly, they received daily oral nimodipine (60 mg every 4 h). Coiling was usually preferred to clipping, except in anatomical conformations that prevented coiling from being used or when intraparenchymal hematoma required surgical evacuation [26]. In the case of hydrocephalus observed on CT, an external ventricular drainage (EVD, Integra Life Science, Saint Priest, France) was inserted. Blood glucose was kept between 8 and 10 mmol/L, systolic arterial pressure between 120 and 160 mmHg before aneurysm repair, SpO_2_ above 96%, and in the case of mechanical ventilation, carbon dioxide partial pressure was kept between 35 and 40 mmHg. An arterial and a central venous catheter were used if patients needed noradrenaline or mechanical ventilation (with or without sedation). 

### 2.5. S100B Measurements

Blood samples were collected at admission and on day 1. They were centrifuged in the biology laboratory and frozen at −20 °C for further analyses. S100B serum levels were measured with an electroluminescent immunoassay kit using a sandwich technique (Cobas, Roche, Mannheim, Germany). This allows the measurement of S100B concentrations between 0.005 and 39 µg/L; the limit of quantification is 0.02 µg/L. The within-assay coefficient of variation (CV) was 2.1% at 0.059 µg/L and 0.9% at 0.372 µg/L mean concentrations. The between-assay CV was 6.2% at 0.200 µg/L and 3.2% at 2.430 µg/L mean concentrations. 

### 2.6. Statistical Analysis

This study follows the standards for reporting diagnostic accuracy studies (STARD) 2015 recommendations for analyses of biomarker diagnostic accuracy [27]. The necessary number of subjects was calculated using the Obuchowski method with the assumption of a ratio of one case (patients with M-GCS < 6 at day 3) to seven controls (patients with M-GCS = 6 at day 3) [28]. A minimum total population of 64 patients, including eight cases (patients with M-GCS < 6 at day 3) was necessary to detect an area under the receiver operating characteristic (ROC) curve (AUC) > 0.8, with a power of 0.8 and an alpha risk of 0.05. 

Data were expressed as their median and interquartile range (IQR). Comparisons between the groups were carried out using the Kruskal–Wallis test and post hoc analyses using the Bonferroni–Dunn test. Comparisons of proportions were made using Fisher’s exact test, with Bonferroni correction for multiple comparisons.

ROC efficiency functions were calculated using the pROC library [29]. The reference standard (M-GCS at day 3) was chosen before the statistical analyses. The best threshold values were calculated according to the point closest to the ROC curve’s top-left point (i.e., optimization of: min ((1 - sensitivity)² + (1-specificity)²). The 95% confidence intervals (95% CI) were calculated using a bootstrapping method. The AUCs were compared using the Delong test. The effect size of the EBI-severe group on S100B levels was evaluated by the Cliff’s delta statistic because of a non-normal distribution (approaching 0 meaning a small effect size and 1 meaning a large effect size) [30]. The AUCs of a combination of EBI markers was evaluated with a logistic regression model, including the following predictors: S100B, SEBES, Hijdra, the presence of an intracerebral hematoma, and GCS at ICU arrival. The dependent variable was the presence or absence of severe EBI at day 3 (i.e., M-GCS < 6).

Biomarkers help to stratify the risk of a condition and guide clinical decision making. Therefore, rather than a single cut-off that dichotomizes the population, another approach is to assess a gray zone with diagnostic uncertainty, allowing for a certain continuum in risk stratification. The first cut-off is chosen to include the diagnosis with near-certainty (i.e., privileging sensitivity); the second is chosen to exclude the diagnosis with near-certainty (i.e., privileging specificity). When the values of the biomarker fall into the gray zone between the two cut-offs, uncertainty exists, and the physician should pursue a diagnosis using additional tools. Therefore, the zone of diagnostic uncertainty, or gray zone, was defined from the first values with a sensitivity or specificity of at least 90% [31].

A value of *p <* 0.05 was considered significant. Statistical analyses were performed using the R software (R Foundation for Statistical Computing, Vienna, Austria, version 3.3.1) [32].

## 3. Results

### 3.1. Population

**Figure 1:**
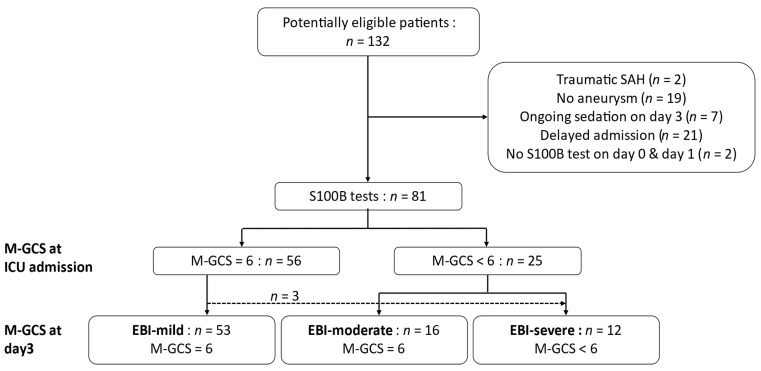
Flow chart. EBI: Early brain injury, GCS: Glasgow coma scale, M-GCS: motor score of GCS, SAH: subarachnoid hemorrhage. Patients’ clinical evolution over the first 3 days and their affiliation to the EBI-mild, EBI-moderate, and EBI-severe groups is presented. Among the three patients who worsened within 3 days, two had hydrocephalus requiring external ventricular drainage (EVD). The third was intubated for respiratory disorder, her M-GCS at day 3 was 5 without sedation and her modified Rankin Scale (mRS) at intensive care unit discharge was 3 (i.e., moderate disability). None had re-bleeding or aneurysm treatment complications.

A total of 132 patients were potentially eligible, and 81 were included in the analyses (Figure 1). For 84% of them, aneurysm was treated by coiling within a median (IQR) of 14 h [5,6,7,8,9,10,11,12,13,14,15,16,17,18,19,20,21]. At ICU admission, 56 patients had an M-GCS of 6, among whom 53 had an M-GCS of 6 both at admission and on day 3; three patients worsened within 3 days (two had hydrocephalus requiring EVD and the third was intubated for respiratory disorder; none had re-bleeding or aneurysm treatment complications). According to the M-GCS at admission and day 3, 53 patients were classified as EBI-mild (M-GCS = 6 at admission and day 3), 16 as EBI-moderate (M-GCS < 6 at admission and M-GCS = 6 at day 3), and 12 as EBI-severe (M-GCS < 6 at day 3 regardless of the M-GCS on admission). In the EBI-severe group, the median M-GCS at day 3 was 4/6 [4,5] (i.e., M-GCS of 5: *n =* 4/12; 4: *n =* 4/12; 2: *n =* 1/12; 1: *n =* 3/12). At day 3, no patients were sedated or had seizures, and all had been treated for hydrocephalus.

The three groups were significantly different in terms of the simplified gravity index (SAPSS), anti-coagulant treatment, WFNS, Fisher grade, Hijdra score, and length of stay, as well as the frequency of hydrocephalus, re-bleeding, and sedation need. Re-bleeding was a rare event (*n =* 4) and occurred within 24 h after admission to the ICU. Patients in the EBI-severe and EBI-moderate groups had a worse outcome at ICU discharge compared to the EBI-mild group (Table 1).

### 3.2. Serum S100B Concentration Changes

We were able to assess the S100B time course in 61 patients with both measurements at admission and on day 1 (EBI-severe: *n =* 11, EBI-moderate: *n =* 13, EBI-mild: *n =* 37). There were no significant differences between admission and day 1 (median (IQR), respectively, 0.098 µg/L (0.067–0.185) vs. 0.100 µg/L (0.069–0.172), *p =* 0.501). Moreover, 46 patients had daily measurements from admission to day 3. The median (IQR) S100B value declined significantly on days 2 (*p =* 0.003) and 3 (*p <* 0.001) compared to admission (admission: 0.094 µg/L (0.061–0.176); day 1: 0.092 µg/L (0.068–0.169); day 2: 0.085 µg/L (0.057–0.112); day 3: 0.064 µg/L (0.048–0.09). 

### 3.3. Accuracy of Serum S100B Concentration to Predict an M-GCS < 6 on Day 3

We included 81 patients with a ≥ 1 S100B measurement at admission or day 1 and used their maximal value to evaluate the S100B diagnostic accuracy to predict a M-GCS < 6 at day 3. The median S100B value was higher in the EBI-severe group (0.467 µg/L, IQR (0.171–1.09)) than in the EBI-moderate (0.134 µg/L, IQR (0.092–0.204); *p =* 0.022) or the EBI-mild group (0.098 µg/L, IQR (0.068–0.138); *p <* 0.001). There was a large effect size of the EBI-severe group vs. the others on the maximal S100B value at admission or day 1, as evidenced by a Cliff’s delta of 0.73 (95% CI (0.46;0.88)). The S100B AUC was not significantly different from that of the GCS at ICU admission (86.7% (95% CI (73.6;95.9) vs. 84.1%, 95% CI (73.9–92.6), *p =* 0.573), and tended to be higher than the AUC of the Hijdra score (68.03% 95% CI (51.4;82.2), *p =* 0.057), as well as the SEBES (68% 95% CI (52.1;81.7), *p =* 0.054). The AUC of a logistic regression model, including S100B, the presence of an intraparenchymal hematoma, the SEBES, the Hijdra score, and the GCS at arrival, was not significantly greater than the AUC of S100B alone (*p =* 0.946).

There were 12 patients with severe EBI and 69 controls (EBI-moderate + EBI-mild), allowing us to detect an AUC > 63% with a power of 95% and an alpha risk of 5%. This value is lower than the lower limit of the 95% CI of the S100B AUC.

Among unconscious patients at admission (i.e., M-GCS < 6, *n =* 25), the effect size of the EBI-severe group on S100B was also large; Cliff’s delta of 0.76 (95%CI (0.34;0.93)). The AUC of S100B (88.2% 95% CI (72.2;100)) was significantly greater than that of the GCS at admission (62.3% (53.1;72.0), *p =* 0.003) and of the Hijdra score (53.8% 95%CI (29.9;77.8), *p =* 0.012), and tended to be better than the AUC of the SEBES (70.1% 95% CI (50;88.5), *p =* 0.151). The AUC of a logistic regression model including S100B, the presence of an intraparenchymal hematoma, the SEBES, the Hijdra score, and the GCS at arrival was not significantly greater than the AUC of S100B alone (*p =* 0.77).

**Figure 2:**
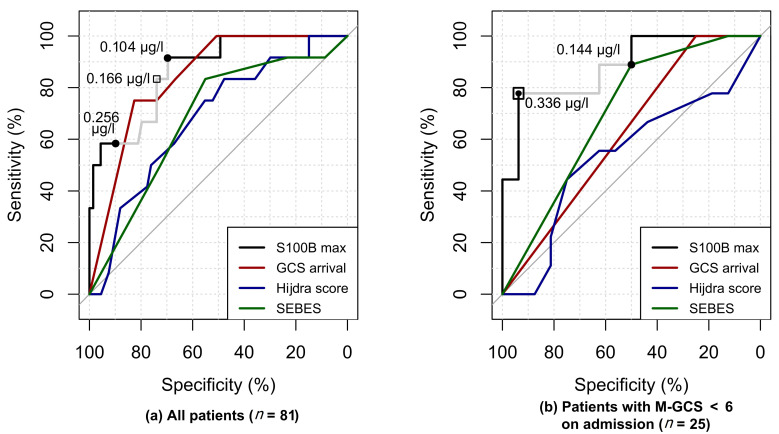
Receiver–operator characteristic (ROC) curves of early brain injury (EBI) biomarkers. **(a)** ROC analyses of the total population (*n =* 81). **(b)** ROC analyses of patients with a motor component of the Glasgow coma score < 6 at admission (*n =* 25). Several S100B thresholds are plotted on both panels: (black square) the best threshold; (gray line) the gray zone of diagnostic uncertainty with (black dots) as its lower limit (i.e., to exclude severe EBI with almost certainty (Se ≥ 90%)) and upper limit (i.e., to predict severe EBI with almost certainty (Sp ≥ 90%)).

The best S100B thresholds and gray zone limits are described in Table 2 and highlighted on the ROC curves in Figure 2.

### 3.4. Serum S100B Concentration and mRS at ICU Discharge

The median maximal serum S100B value at admission or day 1 was significantly different between the seven levels of the mRS (*p <* 0.001). Post hoc analyses found that median S100B values were significantly higher in patients with severe disability (mRS = 5, 0.302 µg/L, IQR (0.216–0.531), *p =* 0.003) or dead (mRS = 6, 1.49 µg/L, IQR (0.957–3.1), *p =* 0.003) compared to those without disability (i.e., mRS < 2, 0.093 µg/L (0.062–0.124)). The maximal serum S100B value at admission or day 1 had an AUC of 95.7% (95% CI (90.52;100)) to predict an mRS > 4 (severe disability or death). A value > 0.262 µg/L (95% CI (0.164;0.494)) predicted an mRS > 4 at ICU discharge with a 94.3% (95% CI (88.6;98.61)) specificity and 81.8% (95% CI (54.6;100)) sensitivity. The gray zone of diagnosis ranged from 0.199 µg/L to 0.262 µg/L. The maximal value of serum S100B was in this gray zone for 12.3% of patients (*n =* 10/81).

Among conscious patients at day 3 (EBI-mild and EBI-moderate groups, *n =* 69), seven had an early S100B > 0.256 µg/L (upper gray zone threshold supposed to predict an M-GCS < 6 with almost certainty), and were considered false positives. They were all Fisher 4 with a SEBES > 2, and three had an intracerebral hematoma. They had a poor outcome (median (IQR) mRS = 4 (3–4.5)) with frequent DCI occurrence (*n =* 5/7, 71.4%).

## 4. Discussion

**Figure 3:**
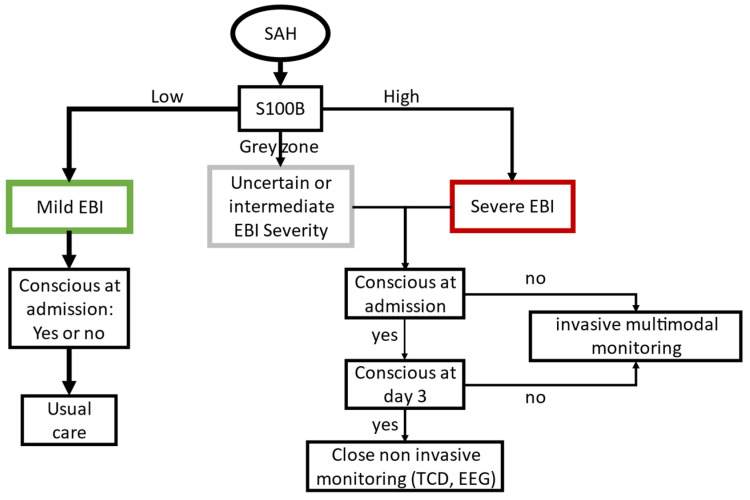
Algorithm chart for early brain injury severity prognostication. Early brain injury (EBI) severity prognostication and the proposed resulting management strategies are presented based on early S100B serum concentration. Low S100B: concentrations below the lower threshold of the gray zone rejecting severe EBI; high S100B: concentrations above the upper threshold of the gray zone predicting severe EBI. An S100B concentration within the gray zone has an uncertain diagnostic value but suggests significant brain injury. EEG: electroencephalography; ICH: intracerebral hematoma; TCD: transcranial doppler.

In this prospective blinded study, the maximal S100B serum concentration at admission and day 1 was found to have a good diagnostic value to predict severe consequences of EBI leading to an unconscious state at day 3 [31]. The early S100B had a better diagnostic accuracy than other EBI markers (i.e., clinical examination, SEBES, or Hijdra scores), in particular for patients unconscious at admission. To stratify the risk of severe EBI, we used a gray zone approach, defined by two thresholds: one to exclude (lower) and one to predict (upper) severe EBI with almost certainty. The lower threshold of the gray zone was close to the 95th percentile reported in healthy controls [33]. Values below this threshold are considered normal by the laboratory (Cobas, Roche, Mannheim, Germany). In the study population, given a 14.8% (*n =* 12/81) pre-test probability of being unconscious at day 3, an S100B level below the gray zone gives a 4% post-test probability, while an S100B level over this gray zone gives a 50% post-test probability (Figure 3). If patients were already unconscious at admission, the pre-test probability was 36% (*n =* 9/25), thus an S100B level below the gray zone gives an 11% post-test probability, while an S100B level above the gray zone gives an 87% post-test probability. Using this approach, patients with an S100B level greater that the upper threshold, but with an M-GCS = 6 at day 3, were considered as false positives. This S100B elevation could, in some patients, be explained by an intraparenchymal hematoma instead of severe EBI. Nevertheless, these patients had a high frequency of DCI and a poor neurological outcome at ICU discharge, suggesting a greater amount of brain injury, despite a reassuring early clinical presentation. Therefore, we propose that patient management strategy and EBI evaluation include an early S100B measurement, in addition to clinical examination and brain imaging. Patients with a low S100B level are expected to have mild EBI with good early and delayed outcomes, while those presenting with moderate or severe EBI are at risk of secondary injury and require close neurological monitoring. Taken together, these results suggest that physicians should adapt their neurological monitoring strategy in patients with moderate or severe EBI without delaying multimodal monitoring (e.g., continuous EEG, transcranial Doppler, or brain oxygen probe, Figure 3).

S100B serum concentration has previously been evaluated to predict long-term prognosis. Since the long-term outcome is the result of early and delayed injury, the peak or mean S100B values of the first week was found to have a good diagnostic value to predict poor long-term outcomes (AUC from 80% to 93%) [18,20,21]. Unlike the values of the first week, early S100B has a poorer diagnostic accuracy to predict a poor outcome (i.e., mRS > 2) [18,21,34]. Herein, S100B was elevated only in patients with an mRS > 4 and was a good predictor of catastrophic outcome, such as death or disorder of consciousness. The early S100B values in patients with intermediate outcomes were more heterogeneous and this could explain why other authors found a poor diagnostic accuracy, as they aimed to predict mRS > 2 (i.e., all levels of disability) [18,21,34], whereas herein the cut-off was mRS > 4 (i.e., severe disability or death).

As evidenced by the sustained S100B elevation, patients with severe EBI undergo prolonged brain aggression leading to catastrophic outcomes. The pathophysiology of these brain injuries is yet to be elucidated, and S100B protein could by itself play a role in brain injury progression. The ratio between serum and cerebral spinal fluid S100B concentration is close to the one early after SAH [35]. Therefore, brain concentration is expected to reach the micromolar range, at which it can have neurotoxic effects [36]. S100B could thus be both a biomarker of brain damage and a damage-associated molecular pattern (DAMP) molecule. There is now growing evidence that DAMPs released upon the primary hemorrhage are involved in EBI and trigger cellular processes, such as neuro-inflammation, eventually leading to delayed ischemia [37]. DAMPs, such as the S100 family of proteins but also HMGB-1 or extracellular matrix-derived proteins, are released into the blood compartment and have been used as biomarkers of cerebral vasospasm, DCI, or long-term outcome [37,38,39]. Furthermore, HMGB-1 early concentration (i.e., at day 1 after the ictus) has been reported to predict the occurrence of cerebral vasospasm regardless of the initial severity [37]. Unlike HMGB-1, early S100B systemic concentration seems to not be predicative of DCI or cerebral vasospasm [21], and further evaluation of several biomarkers of EBI, including DAMPs and markers of cellular damage, are required to identify patient profiles.

Several limitations of this study must be noted. First, there were some missing measurements at admission or day 1. Nevertheless, even if S100B is known to have a short half-life both in the blood and brain extracellular space [40], we and others have found a sustained plasmatic elevation over the first 48 h following the ictus [18,20]; we therefore believe that a single measurement of S100B within the first day is representative of this period. Second, although the number of patients included was higher than that required according to power calculations, the small number of unconscious patients at admission limits the extrapolation of the results to all poor-grade SAH. This preliminary study will need an independent validation cohort to confirm the accuracy of the different thresholds that we suggest to be used at the bedside, in particular among patients with an M-GCS < 6 at admission. Furthermore, we could not separately analyze patients with early complications, such as re-bleeding, in whom the S100B levels did not only reflect brain injury due to the primary hemorrhage. Third, the preference for coiling whenever possible in our institution may also limit the generalizability of the reported thresholds to institutions with a higher frequency of surgical procedures which are known to increase S100B levels [20]. With such a strategy, S100B values are expected to be higher even in those with mild or moderate EBI, and its diagnostic accuracy might thus be poorer with higher thresholds. Finally, since S100B is also expressed in melanocytes and Langerhans cells [41], its serum concentration is known to be higher in dark-skinned people, as well as after sun exposure [42,43]. Although we did not collect patient skin pigmentation, the population admitted to our institution is predominantly Caucasian. 

## 5. Conclusions

This study provides evidence that S100B serum concentration within the first 24 h seems to have a good diagnostic value to predict severe EBI consequences, especially in patients unconscious at admission. Before claiming that S100B could thus be used as a surrogate for EBI severity to start earlier multimodal monitoring, these results must be confirmed in an independent validation cohort.

## Figures and Tables

**Table 1:** Patient characteristics according to EBI group.

	All (*n =* 81)	EBI-Mild (*n =* 53)	EBI-Moderate (*n =* 16)	EBI-Severe (*n =* 12)	*p*-Value
**Demographic data**					
**Median age, years (IQR)**	55 (46–63)	52 (45–60) *	58 (49–65)	61 (57–72)	0.018
**Sex, male, *n* (%)**	29 (36%)	23 (43%)	4 (25%)	2 (17%)	0.141
**Median SAPSS, (IQR)**	25 (19–39)	22 (17–27) ***	41 (28–54) ¥¥¥	58 (39–71)	<0.001
**Aneurysm characteristics**					
**Median size, mm (IQR)**	4.8 (3.5–7.7)	4.7 (3.6–7.6)	4.5 (3.3–8.8)	4.8 (4–6.7)	0.987
**Location, *n* (%)**					
anterior circulation (ACA, ACoA, AChA, pericalosal)	29 (36%)	21 (40%)	2 (13%)	6 (50%)	0.094
**posterior circulation (PCA, PCoA)**	8 (10%)	5 (9%)	2 (13%)	1 (8%)	0.851
**MCA**	21 (26%)	14 (26%)	5 (31%)	2 (17%)	0.588
**carotid**	5 (6%)	4 (8%)	1 (6%)	0	1
**vertebrobasilary**	14 (17%)	8 (15%)	4 (25%)	2 (17%)	0.496
**Hydrocephalus, *n* (%)**	30 (37%)	11 (21%)	12 (75%) ¥¥¥	7 (58%)	<0.001
**Sedation at admission, *n* (%)**	23 (28%)	2 (4%) ***	12 (75%) ¥¥¥	9 (75%)	<0.001
**Re-bleeding, *n* (%)**	4 (5%)	0 **	1 (6%)	3 (25%)	0.003
Aneurysm treatment, number of coiling, *n* (%)	68 (84%)	47 (89%)	13 (81%)	8 (67%)	0.134
Median time from admission to treatment, h (IQR)	14 (5–21)	14 (8–20)	14 (3–21)	43 (15–68)	0.150
**Aneurysm treatment complication, *n* (%)**	15 (19%)	8 (15%)	5 (31%)	2 (17%)	0.259
WFNS at first physician contact, *n* (%)					
**1–2**	55 (68%)	46 (87%) **	5 (31%) ¥¥¥	4 (33%)	<0.001
**3–5**	26 (32%)	7 (13%) **	11 (69%) ¥¥¥	8 (67%)	<0.001
**Fisher grade, *n* (%)**					
**1–2**	8 (10%)	8 (15%)	0	0	0.158
**3–4**	73 (90%)	45 (85%)	16 (100%)	12 (100%)	0.158
Median Hijdra score (IQR)	18 (12.5–21.5)	15 (10–19.5) **	20 (19;22.5) ¥	20.5 (18.5;23)	<0.001
**SEBES > 2, *n* (%)**	64 (79%)	39 (73.6%)	14 (87.5%)	11 (91.7%)	0.305
**ICH, *n* (%)**	17 (21%)	8 (15.1%)	4 (25%)	5 (41.7%)	0.107
Median GCS at ICU admission, (IQR)	14 (3–15)	15 (14–15)	3 (3–4)	3 (3–6)	<0.001
**DCI occurrence, *n* (%)**	20 (25%)	10 (19%)	7 (44%)	3 (25%)	0.116
Median mRS at ICU discharge (IQR)	3 (1–4)	2 (1–3)***	3 (3–4) *, ¥	5 (5–6)	<0.001
Median length of ICU stay, days (IQR)	11 (9–16)	10 (9–13)	16 (13–18) ¥¥	11 (4–18)	0.012

The three groups were defined according to M-GCS at ICU admission and day 3: EBI-mild M-GCS = 6 at arrival and day 3, EBI-moderate M-GCS < 6 at arrival and =6 on day 3, EBI-severe M-GCS < 6 both at arrival and day 3. Results of post hoc tests: Differences compared to the EBI-severe group, * *p <* 0.05 ** *p <* 0.01 *** *p <* 0.001; differences between EBI-moderate and EBI-mild groups, ¥ *p <* 0.05 ¥¥ *p <* 0.01 ¥¥¥ *p <* 0.001. GCS: Glasgow coma scale, OAD: oral antidiabetic drug, ACA: anterior cerebral artery, ACoA: anterior communicative artery, AChA: choroidal artery, PCA: posterior cerebral artery, PCoA: posterior communicative artery, MCA: mean cerebral artery, DCI: delayed cerebral ischemia, ICH: intracerebral hematoma; mRS: modified Rankin Scale.

**Table 2:** Accuracy of maximal S100B and GCS at ICU admission to predict an M-GCS < 6 on day 3 in the whole cohort and only in patients with an M-GCS < 6 at ICU admission.

	All Patients (*n =* 81, M-GCS < 6 at Day 3 *n =* 12)		Patients with M-GCS < 6 at Admission (*n =* 25, M-GCS < 6 at Day 3 *n =* 9)	
	S100B Max	GCS at Admission	S100B Max	GCS at Admission
AUC, % (95% CI)	86.7 (73.6;95.9)	84.1 (73.9–92.6)	88.2 (72.2;100)	62.5 (53.1;72.0)
Best threshold (95% CI)	0.165 µg/L (0.100;0.494)	4.5 (4.5–14.5)	0.336 µg/L (0.123;0.951)	4.5 (4.5;8.5)
Se, % (95% CI)	83.3 (58.3;100)	75.0 (50;100)	77.7 (44.4;100)	100
Sp, % (95% CI)	73.9 (62.3;84.1)	82.6 (73.9;91.3)	93.8 (81.3;100)	25 (6.3;43.8)
PLR (95% CI)	3.2 (2.0;5.1)	4.3 (2.3;7.9)	12.4 (1.8;85.7)	1.3 (0.9–1.8)
NLR (95% CI)	0.23 (0.06;0.81)	0.30 (0.11;0.81)	0.24 (0.07;0.81)	0.19 (0.01;3.14)
Gray zone µg/L	0.104–0.256	13.5–4.5	0.144–0.336	4.5–3
Inter-LHR	0.16–5.75	0.25–4.32	0.22–12.4	1.2–0.19
% in gray zone	38	14	32	84

The gray zones of diagnostic uncertainty are presented with the percentage of patients inside, and their interval likelihood ratio (inter-LHR). The positive LHR of the highest value of the biomarker in the gray zone is considered to include the diagnosis and the negative LHR of the lowest value to exclude the diagnosis. AUC: area under the curve, 95% CI: 95% confidence interval, Se: sensitivity, Sp: specificity, PLR: positive likelihood ratio, NLR: negative likelihood ratio.
